# A Case of Gastric Neuroendocrine Tumor With a Raspberry‐Like Appearance on the Background of Acid‐Suppressive Therapy‐Related Gastropathy

**DOI:** 10.1002/deo2.70309

**Published:** 2026-03-02

**Authors:** Yoshiaki Moriguchi, Jun Nakahodo, Mari Iseki, Toshihiro Funabiki, Yasuhiro Oka, Eriko Noma, Takeo Arakawa, Shin‐ichiro Horiguchi, Osamu Goto, Toshiro Iizuka

**Affiliations:** ^1^ Department of Gastroenterology, Tokyo Metropolitan Cancer and Infectious Diseases Center Komagome Hospital Tokyo Japan; ^2^ Department of Endoscopy, Tokyo Metropolitan Cancer and Infectious Diseases Center Komagome Hospital Tokyo Japan; ^3^ Department of Pathology, Tokyo Metropolitan Cancer and Infectious Diseases Center Komagome Hospital Tokyo Japan

**Keywords:** endoscopic submucosal dissection, gastric neuroendocrine tumor, gastric tumor with a raspberry‐like appearance, PPI/p‐CAB–related gastropathy, vonoprazan

## Abstract

We report the case of a 52‐year old man who was referred to our institution after a routine health check identified an asymptomatic gastric polyp. The medical history of the patient included reflux esophagitis, for which he had been treated with vonoprazan 10 mg daily for 6 years after initially receiving proton pump inhibitors. Endoscopy revealed a 4‐mm, hemispherical, uniformly reddish Yamada type II lesion on the anterior wall of the gastric body, arising from nonatrophic mucosa with multiple small hyperplastic polyps. Magnifying narrow‐band imaging revealed a regular microvascular and microsurface pattern, producing a raspberry‐like appearance. Histological examination of the biopsy specimen revealed a grade 1 neuroendocrine tumor. Contrast‐enhanced computed tomography revealed no evidence of metastatic lesions. En bloc resection was successfully performed using endoscopic submucosal dissection. Pathological examination confirmed a 5‐mm grade 1 neuroendocrine tumor with negative margins and no vascular invasion. Gastric neuroendocrine tumors with raspberry‐like morphology are rare. This case underscores the importance of considering gastric neuroendocrine tumors in the differential diagnosis of raspberry‐like gastric lesions, particularly in patients with potent acid‐suppressive therapy–related gastropathy.

**Trial Registration**: N/A.

## 1 Introduction

The endoscopic and clinicopathological features of Rindi classification Type I and Type III gastric neuroendocrine tumors have been well described. In contrast, those of the proposed acid‐suppression–associated subtype remain unclear [1]. Gastric neuroendocrine tumors resembling a raspberry‐like lesion are rare. To our knowledge, this is the first reported case of a gastric neuroendocrine tumor with raspberry‐like morphology in a patient with proton pump inhibitors (PPIs) and potassium‐competitive acid blocker (p‐CAB)‐related gastropathy [2, 3]. This report highlights the importance of recognizing atypical endoscopic appearances that may mimic raspberry‐like gastric lesions to ensure accurate diagnosis and management.

## 2 Case Report

**FIGURE 1:**
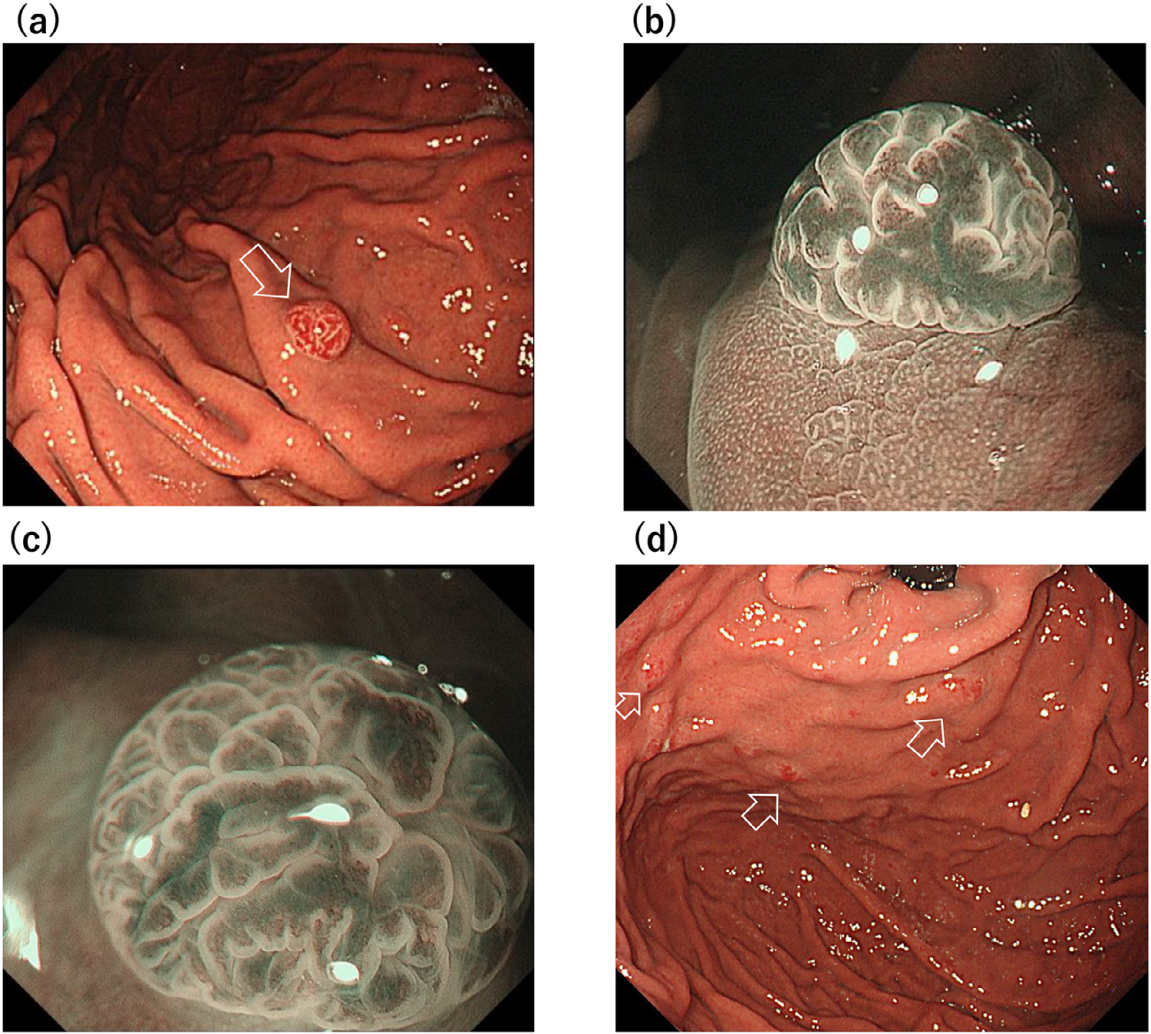
Endoscopic findings. (a) White‐light endoscopy showing a bright red hemispherical lesion on the anterior gastric body wall. (b, c) Magnified narrow‐band imaging (NBI) reveals a relatively regular gyri‐like surface pattern with dilated, tortuous vessels within widened interpit areas, without prominent tree‐like vascular changes. (d) Absence of atrophy in the background gastric mucosa and multiple small hyperplastic polyps consistent with potent acid‐suppressive therapy‐related gastropathy.

**FIGURE 2:**
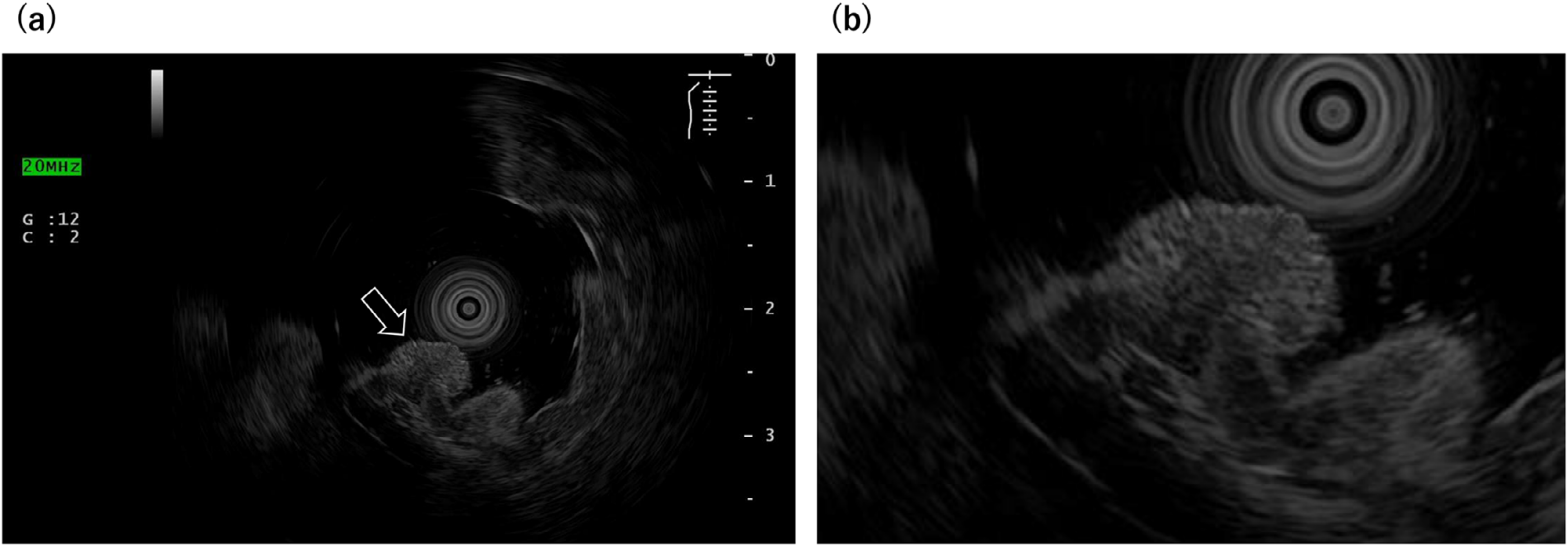
Endoscopic ultrasonography (EUS). (a) A hypoechoic, 4‐mm lesion localized to the second layer of the gastric wall. (b) The third layer remained intact, with no thinning or disruption, indicating the confinement of the lesion.

**FIGURE 3:**
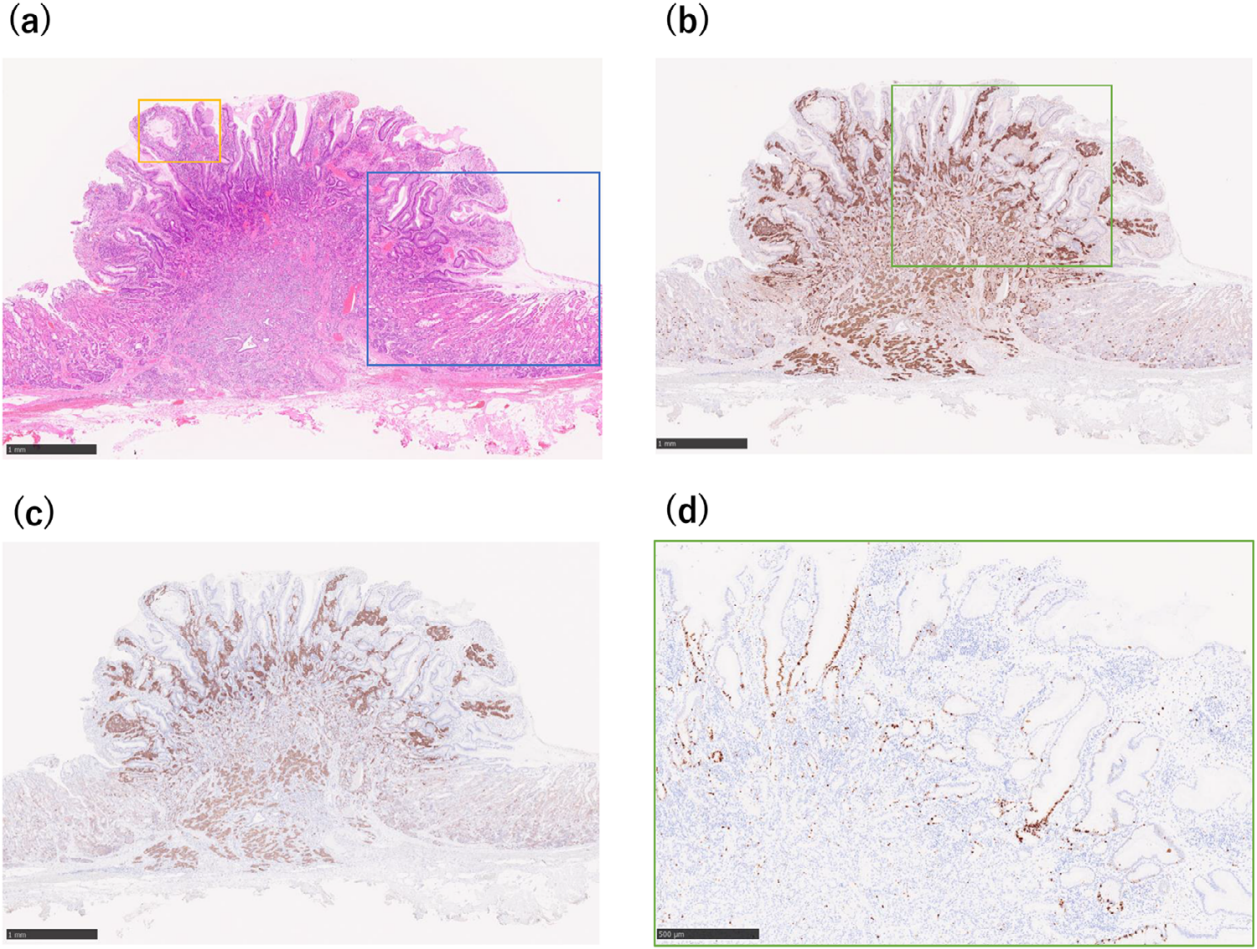
Histopathological findings. (a) Hematoxylin and eosin staining showing nests and ribbons of uniform tumor cells within the lamina propria and submucosa. (b) Chromogranin A immunostaining highlights tumor cells extending to the mucosal surface. (c) Synaptophysin immunostaining positive in tumor cells. (d) Higher‐magnification view of the boxed area in (b), showing MIB‐1 (Ki‐67) immunostaining with a labeling index of 1.5%, consistent with a grade 1 neuroendocrine tumor.

**FIGURE 4:**
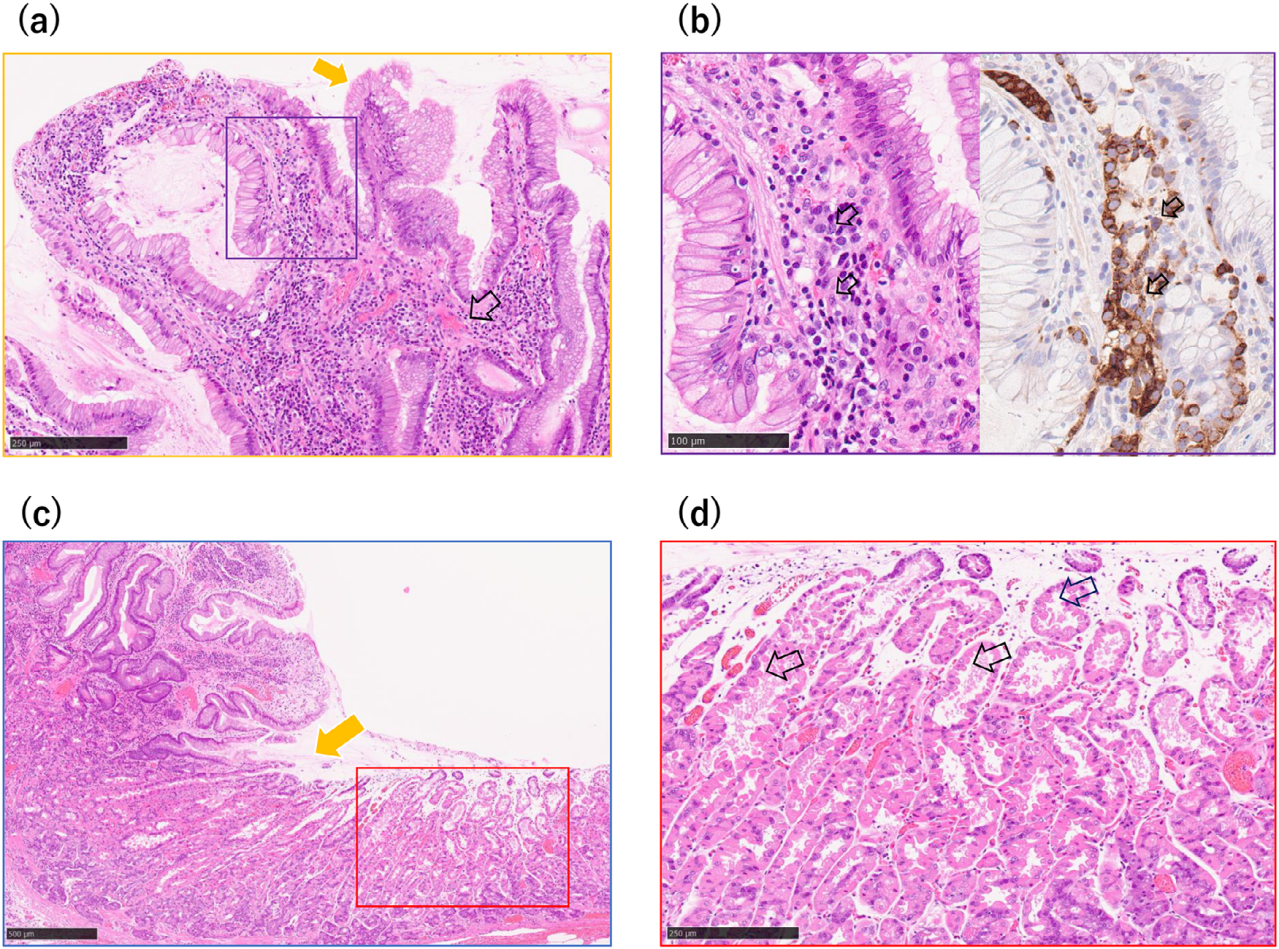
Higher‐magnification views of mucosal and background changes. (a) Higher‐magnification view of the yellow boxed area in Figure 3a, showing overlying foveolar epithelium with hyperplastic changes and dilated capillaries, with tumor cells extending to the mucosal surface, corresponding to the raspberry‐like appearance. (b) Higher‐magnification view of the purple boxed area in Figure 4a with Synaptophysin. Neuroendocrine tumor extends to the mucosal surface. (c) Higher‐magnification view of the blue boxed area in Figure 3a, demonstrating a well‐demarcated tumor margin and background mucosa with dilated fundic glands. (d) Higher‐magnification view of the red boxed area in Figure 4c, showing background oxyntic mucosa with parietal cell protrusions into the glandular lumen without evidence of enterochromaffin‐like (ECL) cell hyperplasia.

A 52‐year old man was referred to our hospital for further evaluation after a gastric polyp was detected during a routine health check. Biopsy confirmed a grade 1 neuroendocrine tumor. His medical history included reflux esophagitis, for which he had been treated with vonoprazan 10 mg daily for 6 years after initially taking PPIs. The social history of the patient was notable for habitual alcohol intake (approximately 1000 mL of 7% alcohol per day) and a 24‐year history of heavy smoking (40 cigarettes per day from 18 to 42 years of age). Fasting serum gastrin concentration, measured while the patient was taking vonoprazan 10 mg daily, was 170 pg /mL (reference range, 11.9–46.9). Other findings included neuron‐specific enolase of 11.8 ng/mL, a negative *Helicobacter pylori* IgG antibody, and negative parietal cell and intrinsic factor antibodies. Endoscopy revealed a 4‐mm bright red, sharply demarcated, hemispherical lesion on the anterior wall of the mid‐gastric body (Figure 1a). Magnifying narrow‐band imaging (NBI) revealed a relatively regular gyri‐like surface pattern with white‐zone thickening and tortuous vessels within the widened interpit areas (Figure 1b,c). The background mucosa was nonatrophic and contained multiple small hyperplastic polyps, consistent with PPI‐related gastropathy (Figure 1d). Endoscopic ultrasonography (EUS) revealed a slightly hypoechoic 4‐mm lesion confined to the second layer, without thinning or disruption of the third layer (Figure 2). Contrast‐enhanced computed tomography showed no evidence of lymph nodes or distant metastasis. In the absence of autoimmune gastritis and Zollinger–Ellison syndrome, the lesion was considered compatible with a sporadic (Type III) gastric neuroendocrine tumor; however, strict classification remained challenging because the lesion arose in the setting of long‐term potent acid‐suppressive therapy. Because the lesion was solitary, <10 mm, preservation of the third layer, and without evidence of metastasis, endoscopic submucosal dissection (ESD) was performed. Histopathological examination revealed tumor cells proliferating in nests and pseudoglandular patterns within the lamina propria and submucosa while preserving the existing glandular architecture (Figure 3a). No mitotic cells were observed. Immunohistochemistry revealed positivity for chromogranin A (Figure 3b) and synaptophysin (Figure 3c), with an MIB‐1 index of 1.5% (Figure 3d), consistent with a grade 1 neuroendocrine tumor. Venous (Elastica van Gieson staining) and lymphatic (D2‐40 immunostaining) invasions were not identified. The final diagnosis was a grade 1 neuroendocrine tumor (5 × 4 mm, ly0, v0, pHM0, and pVM0). Hyperplastic changes were observed in the overlying foveolar epithelium, with tumor cells extending to the mucosal surface (Figure 4a,b). The background mucosa showed parietal cell protrusion but no enterochromaffin‐like (ECL) cell hyperplasia (Figure 4c,d).

## 3 Discussion

This report describes a rare case of a gastric neuroendocrine tumor with a raspberry‐like appearance.

Rindi Type I gastric neuroendocrine tumors are typically reddish and polypoid, whereas Type III tumors more often present with a submucosal tumor–like appearance [4]. Raspberry‐like gastric lesions exhibit overlapping endoscopic features across multiple entities. On magnifying NBI, white‐zone thickening reflects hyperplastic foveolar epithelium and is a characteristic feature of hyperplastic polyps in *H. pylori*‐uninfected stomachs. In contrast, foveolar‐type gastric adenomas typically exhibit papillary or gyrus‐like microstructures with a clear demarcation line and usually lack white‐zone thickening. The homogeneous bright‐red color, gyrus‐like surface, and thick white zone in this case resembled a hyperplastic polyp. Fundic gland–type gastric carcinoma may mimic a raspberry‐like surface but typically appears flatter and shows subtle vascular irregularities, as summarized in Table S1 [4, 5, 6]. Previously reported gastric neuroendocrine tumors associated with long‐term acid‐suppressive therapy occurred after at least 4 years of PPI use. Regarding macroscopic morphology, six lesions were described as submucosal tumor–like and four as polypoid. Among cases with available data, serum gastrin levels were reported as elevated or mildly elevated in the majority, and submucosal tumor–like morphology was frequently observed, as summarized in Table S2 [2, 7, 8]. However, detailed descriptions of surface structures, including magnifying endoscopic findings, remain limited. In contrast, the present case demonstrated a small polypoid lesion with a distinctive raspberry‐like surface appearance during long‐term p‐CAB therapy. Magnifying NBI showed a relatively regular gyri‐like surface pattern with white‐zone thickening and tortuous vessels within widened interpit areas, highlighting a unique endoscopic presentation.

Several mechanisms may explain this epithelial hyperplasia. As discussed by Chetty et al., foveolar epithelial hyperplasia and neuroendocrine cell proliferation may represent parallel responses to a shared background condition, such as hypergastrinemia associated with chronic gastric mucosal injury. Preferential localization of neuroendocrine components within hyperplastic mucosa suggests that the local mucosal microenvironment may facilitate neuroendocrine proliferation. Paracrine interactions between hyperplastic epithelium and neuroendocrine cells have also been proposed [9].

In the present case, histopathology showed a well‐differentiated gastric neuroendocrine tumor extending to the mucosal surface. Tumor‐associated angiogenesis, suggested by increased capillary density, may provide excess trophic support to the surrounding foveolar epithelium. The patient also exhibited hypergastrinemia associated with long‐term vonoprazan use. Gastrin stimulates ECL cell growth and foveolar epithelial proliferation, and systemic hypergastrinemia may have further exacerbated epithelial hyperplasia. These mechanisms may act synergistically to produce the distinctive raspberry‐like morphology, underscoring the diagnostic challenge posed by superficial presentations.

Gastric neuroendocrine tumors are typically visualized on EUS as hypoechoic lesions arising from the second to third layers of the gastric wall [10]. In the present case, EUS demonstrated a small hypoechoic lesion confined to the second layer with preservation of the third layer. Although EUS has limitations in distinguishing gastric neuroendocrine tumors from other submucosal tumors (e.g., gastrointestinal stromal tumors and leiomyomas) based on echogenicity alone, it is useful for assessing invasion depth and supporting treatment planning. These findings supported the decision to perform ESD. Among the previously reported cases and the present case, four patients underwent surgical resection, and five were treated endoscopically; in one case, the tumor regressed after discontinuation of PPI therapy (Table S2). As reported by Egbert et al., acid‐suppression–associated gastric neuroendocrine tumors appear to have low malignant potential, with extremely low rates of lymph node and distant metastasis and favorable disease‐specific outcomes [1]. Most tumors are small, low‐grade lesions arising in nonatrophic oxyntic mucosa, and recurrence, when present, is typically confined to the stomach. In their cohort, most patients were successfully managed endoscopically, and surgery with lymphadenectomy was rarely required [1]. Accordingly, complete en bloc endoscopic resection appears to be an appropriate approach in selected patients, and follow‐up strategies should be individualized.

P‐CABs exert more potent and sustained acid suppression than PPIs and may induce more profound hypergastrinemia, potentially influencing tumor development. Among reported cases of gastric neuroendocrine tumors associated with long‐term acid‐suppressive therapy, only one involved p‐CAB exposure (Table S2), and retrospective review suggested that the lesion predated initiation of p‐CAB therapy [2]. Thus, although p‐CAB therapy may exacerbate hypergastrinemia, its role in gastric neuroendocrine tumor development and recurrence remains uncertain. Discontinuation of acid‐suppressive therapy may be considered when clinically feasible, as one case demonstrated complete tumor regression after PPI discontinuation alone [7].

With the increasing use of PPIs and p‐CABs, this case describes a rare morphological presentation of gastric neuroendocrine neoplasms that may be associated with hypergastrinemia. Further accumulation of similar cases and systematic investigation is required to clarify the clinical significance of such findings.

## Author Contributions

**Yoshiaki Moriguchi**: conceptualization, investigation, and writing – original draft. **Jun Nakahodo**: supervision, validation, and writing – review & editing. **Mari Iseki**: investigation, resources, and visualization. **Toshihiro Funabiki**: investigation, resources, and visualization. **Yasuhiro Oka**: investigation, resources, and visualization. **Eriko Noma**: investigation, resources, and visualization. **Takeo Arakawa**: investigation, resources, and visualization. **Shin‐ichiro Horiguchi**: investigation, resources, and visualization. **Osamu Goto**: investigation, resources, and visualization. **Toshiro Iizuka**: supervision, project administration, and writing – review & editing. All authors have reviewed and approved the final version of the manuscript.

## Conflicts of Interest

The authors declare no conflicts of interest.

## Funding

None.

## Ethics Statement

**Approval of the research protocol by an Institutional Review Board: N/A**. The authors report the details of this patient's case in accordance with the ethical standards of the Declaration of Helsinki.

## Consent

Written informed consent was obtained from the patient for publication of this case report and accompanying images.

## Supporting information




**TABLE S1** Endoscopic characteristics of gastric NETs and raspberry‐like gastric lesions.

**TABLE S2** Previously reported gastric neuroendocrine tumors associated with acid‐suppressive therapy.
