# Supplementary material for: A Case of Gastric Neuroendocrine Tumor With a Raspberry‐Like Appearance on the Background of Acid‐Suppressive Therapy‐Related Gastropathy
Source: DEN Open. 2026 Mar 2;6(1):e70309. doi: 10.1002/deo2.70309 (PMC12953051; doi:10.1002/deo2.70309)
Supplement: Supplementary file 1 — TABLE S1 Endoscopic characteristics of gastric NETs and raspberry‐like gastric lesions. [file DEO2-6-e70309-s001.docx]

**Supplementary Table 1. Endoscopic characteristics of gastric NETs and raspberry-like gastric lesions**

| **Entity** | **Typical clinical background** | **WLI findings** | **Magnifying NBI findings** | **Key differentiating points** |
| --- | --- | --- | --- | --- |
| **Gastric NET Type I** | Autoimmune gastritis, hypergastrinemia | Multiple, small, red polypoid lesions in gastric body/fundus; with central depression | Enlarged groove-type mucosal structure with dilated surface vessels; absent MS plus irregular MV in depression | Usually multiple; background atrophic gastritis |
| **Gastric NET Type III** | Sporadic; normal gastrin level | Solitary, larger lesion; often isochromatic SMT-like; possible ulceration | Regular MS plus MV without demarcation line; sometimes unclassifiable | More aggressive; often larger and solitary |
| **PPI-associated subtype Gastric NET** **(proposed Type IV)** | Long-term PPI/PCAB use; hypergastrinemia without autoimmune gastritis | Small, body-predominant, often multiple; polypoid or SMT-like lesions | Limited data | Clinical phenotype resembles Type I/II; detailed morphological classification remains unclear in literature |
| **Foveolar-type gastric adenoma (FGA)** | Hp-naïve stomach; often PPI/PCAB users | Small (<5 mm), bright-red, granular-surfaced, raspberry-like polyp | Papillary or gyrus-like microstructure; visible capillaries; clear demarcation line; absence of white-zone thickening | Bright red color, papillary/gyrus-like surface, sharp demarcation; mimics hyperplastic polyp but lacks white-zone thickening |
| **Gastric hyperplastic polyp (Hp-naïve)** | Hp-naïve; post-acid suppression | Small, reddish, raspberry-like protruded lesion | Tubular-dominant microstructure; white-zone thickening; indistinct or partial demarcation | White-zone thickening reflects foveolar hypertrophy; less distinct demarcation than FGA |
| **Fundic gland–type gastric carcinoma (FG-type GC)** | Hp-naïve stomach; often asymptomatic | Flat or slightly elevated lesion, pale to light-reddish; sometimes raspberry-like surface | Regular or slightly irregular microstructure; subtle vascular changes; often covered by non-neoplastic foveolar epithelium | Flat morphology, mild surface irregularity; invasive component may be underestimated endoscopically |

**Abbreviations**

ECL cell: enterochromaffin-like cell

FGA: foveolar-type gastric adenoma

FG-type GC: fundic gland–type gastric carcinoma

Hp: Helicobacter pylori

Hp-naïve: Helicobacter pylori–uninfected

MS: microsurface

MV: microvascular

NBI: narrow-band imaging

NET: neuroendocrine tumor

p-CAB: potassium-competitive acid blocker

PPI: proton pump inhibitor

SMT: submucosal tumor

WLI: white-light imaging
