# Supplementary material for: A Case of Gastric Neuroendocrine Tumor With a Raspberry‐Like Appearance on the Background of Acid‐Suppressive Therapy‐Related Gastropathy
Source: DEN Open. 2026 Mar 2;6(1):e70309. doi: 10.1002/deo2.70309 (PMC12953051; doi:10.1002/deo2.70309)
Supplement: Supplementary file 2 — TABLE S2 Previously reported gastric neuroendocrine tumors associated with acid‐suppressive therapy. [file DEO2-6-e70309-s002.docx]

**Supplementary table 2. Previously reported gastric neuroendocrine tumors associated with acid-suppressive therapy**

| **Author (Year)** | **Age / Sex** | **Acid-suppressive therapy** | **Duration** | **Serum gastrin** | **Background mucosa** | **Tumor size** | **Location** | **Morphology** | **Histology / Grade** | **Background mucosa** | **H. pylori** | **Treatment** |
| --- | --- | --- | --- | --- | --- | --- | --- | --- | --- | --- | --- | --- |
| Dawson et al. (2000) | 56 /F | Long-term PPI | 4 yrs | 128 pmol/L | Non-atrophic gastric mucosa | 20 mm | Gastric body | SMT-like | Gastric carcinoid | Non-atrophic gastric mucosa | No detail | Surgery |
| Jianu et al. (2012) – Pt 1 | 55 / M | Lansoprazole 30 mg daily → Esomeprazole 40 mg daily | 12 yrs | Not assessed on PPI | Oxyntic mucosa normal / no gastritis、ECL cell hyperplasia | 18 mm | Upper gastric body | SMT-like | NET G1 (Ki-67 <2%) | Oxyntic mucosa normal / no gastritis、ECL cell hyperplasia | Negative | Endoscopic resection |
| Jianu et al. (2012) – Pt 2 | 68 / F | Lansoprazole 30 mg daily → Pantoprazole 40 mg daily | 13 yrs | 191 pmol/L (on PPI) | Flat oxyntic mucosa: no atrophic gastritis、parietal cells abundant、linear & micronodular ECL hyperplasia | 15 mm | Proximal gastric body | SMT-like | Well-differentiated NET / G1、Ki-67 <2% | Flat oxyntic mucosa: no atrophic gastritis、linear & micronodular ECL hyperplasia | Positive | PPI cessation → tumor disappeared by 3 months |
| Jianu et al. (2012) | 49 / M | Lansoprazole 30 mg daily | 15 yrs | 159 pmol/L | Endoscopically normal oxyntic mucosa: ECL cell hyperplasia | ~20 mm | Hiatal hernia | SMT-like | Poorly differentiated NEC、Ki-67 ~90% | Endoscopically normal oxyntic mucosa: ECL cell hyperplasia | Negative | Surgery |
| Lahner et al. (2014) | 48 / F | PPI | 8 yrs | Normal | Mild chronic gastritis of corpus, no atrophy/intestinal metaplasia; ECL hyperplasia | 10 mm | Cardia | Polypoid | NET G1 (Ki-67 1%) | Mild chronic gastritis of corpus, no atrophy/intestinal metaplasia | Negative | EMR |
| Cavalcoli et al. (2015) – Pt 1 | 76 / M | PPI | ≥15 yrs | 1250 pg/mL | Moderate chronic antral gastritis without atrophy; ECL-cell hyperplasia（fundus） | 4 mm | Fundus | Polypoid | NET G1 (Ki-67 <1%) | Moderate chronic antral gastritis without atrophy; ECL-cell hyperplasia | Negative | Endoscopic resection / polypectomy |
| Cavalcoli et al. (2015) – Pt 2 | 58 / M | PPI | 5 yrs | 88 pg/mL | Mild chronic inflammation in corpus without atrophy; linear ECL-cell hyperplasia; cystic gland polyps | 5 mm | Gastric body | Polypoid | NET G2 (Ki-67 4%) | Mild chronic inflammation in corpus without atrophy; linear ECL-cell hyperplasia | Negative | Endoscopic resection/ polypectomy |
| Cavalcoli et al. (2015) – Pt 3 | 67 / F | PPI | ≥15 yrs | 136 pg/mL | moderate chronic antral gastritis without atrophy; corpus mild chronic inflammation without atrophy） | 18 mm | Fundus | SMT-like | NET G1(Ki-67 <2%) | Non-atrophic corpus mucosa | Negative | Total gastrectomy |
| Nandy et al. (2016) | 78 / M | Omeprazole 20 mg twice daily | >20 yrs | 657 pg/mL | No H. pylori-associated or other gastritis（adjacent mucosa） | 30 mm | Gastric body | SMT-like | NET G1 | No gastritis（adjacent mucosa） | Negative | Laparoscopic wedge resection |
| Nagao et al.  (2024) | 52 /M | Lansoprazole → vonoprazan | 14 yrs | 1030 pg/mL | No atrophic changes in the fundic gland  mucosa; linear ECL-cell hyperplasia | 9mm | Gastric body | Polypoid | NET G1 (Ki-67 <1%) | No atrophic changes in the fundic gland  mucosa; linear ECL-cell hyperplasia | Positive  (after eradication) | ESD |
| Present case | 52 /  M | Vonoprazan 10 mg daily 6 years (after prior PPIs) | 6 yrs | 170 pg/mL (on-therapy) | Non-atrophic oxyntic mucosa with foveolar hyperplasia; parietal cell | 5 mm | Gastric body | Polypoid | NET G1 | Non-atrophic oxyntic mucosa with parietal cell hyperplasia | Negative | ESD |

Abbreviations

ECL: enterochromaffin-like

EMR: endoscopic mucosal resection

ESD: endoscopic submucosal dissection

HP: Helicobacter pylori

NBI: narrow-band imaging

NEC: neuroendocrine carcinoma

NET: neuroendocrine tumor

p-CAB: potassium-competitive acid blocker

PPI: proton pump inhibitor

SMT: submucosal tumor
